# Quantification of stroke lesion volume using epidural EEG in a cerebral ischaemic rat model

**DOI:** 10.1038/s41598-021-81912-2

**Published:** 2021-01-27

**Authors:** Hyun-Joon Yoo, Jinsil Ham, Nguyen Thanh Duc, Boreom Lee

**Affiliations:** 1 Department of Physical Medicine and Rehabilitation, Korea University Anam Hospital, Korea University College of Medicine, 73 Goryeodae-ro, Seongbuk-gu, Seoul, 02841 Korea; 2 Department of Biomedical Science and Engineering (BMSE), Institute Integrated Technology (IIT), Gwangju Institute of Science and Technology (GIST), 123 Cheomdan-gwagiro, Buk-gu, Gwangju, 61005 Korea

**Keywords:** Stroke, Stroke

## Abstract

Precise monitoring of the brain after a stroke is essential for clinical decision making. Due to the non-invasive nature and high temporal resolution of electroencephalography (EEG), it is widely used to evaluate real-time cortical activity. In this study, we investigated the stroke-related EEG biomarkers and developed a predictive model for quantifying the structural brain damage in a focal cerebral ischaemic rat model. We enrolled 31 male Sprague–Dawley rats and randomly assigned them to mild stroke, moderate stroke, severe stroke, and control groups. We induced photothrombotic stroke targeting the right auditory cortex. We then acquired EEG signal responses to sound stimuli (frequency linearly increasing from 8 to 12 kHz with 750 ms duration). Power spectral analysis revealed a significant correlation of the relative powers of alpha, theta, delta, delta/alpha ratio, and (delta + theta)/(alpha + beta) ratio with the stroke lesion volume. The auditory evoked potential analysis revealed a significant association of amplitude and latency with stroke lesion volume. Finally, we developed a multiple regression model combining EEG predictors for quantifying the ischaemic lesion (R^2^ = 0.938, *p* value < 0.001). These findings demonstrate the potential application of EEG as a valid modality for monitoring the brain after a stroke.

## Introduction

Stroke is a neurological disorder caused by vascular disease, including cerebral infarction, intracerebral haemorrhage, and subarachnoid haemorrhage^1^. It is the second leading cause of death worldwide and its treatment incurs a substantial economic cost^2^. Consequently, comprehensive clinical guidelines for stoke management have been complied as countermeasures for reducing stroke-related disabilities^3^. However, the global burden of stroke remains high^2^. Moreover, stroke is a heterogeneous condition and its clinical course is difficult to predict. Further, there may be progressive stroke-induced brain damage during the subacute stage, which further aggravates the neurological outcome^4^. Therefore, precise monitoring and evaluation of the brain injury are paramount for establishing effective treatment strategies and prognosis prediction.

With advances in modern medicine, various efficient imaging modalities and evaluation tools have been developed to monitor and evaluate the post-stroke brain condition. Among them, computed tomography (CT) and magnetic resonance imaging (MRI) are the most widely used modalities due to their sensitivity to brain ischaemic or haemorrhagic changes and their provision of structural information^5^. Therefore, it is recommended that patients admitted to hospitals with suspected stroke undergo immediate brain imaging evaluation^3^. However, these neuroimaging modalities are expensive and may not be available in some facilities. Therefore, the use of CT or MRI for repetitive monitoring is limited despite being the diagnostic tools of choice for stroke detection. However, although these imaging modalities provide detailed anatomical information about the brain; they do not assess the functional status of the brain. There are other alternative imaging techniques such as positron emission tomography (PET), which is often used to evaluate brain metabolism. However, the associated radiation exposure and high costs limit its feasibility as a routine clinical monitoring tool^6^. Stroke-related clinical scales such as the National Institutes of Health Stroke Scale (NIHSS) and the Mini-Mental State Examination (MMSE) are also widely used^7,8^. Although the administration of these scales requires well-trained physicians, they are relatively simple to implement and have been reported useful for estimating the neurologic deficit changes in patients over time^9^. However, they require appropriate cooperation from patients and cannot be administered to patients who are aphasic or under anaesthesia. Additionally, clinical scales are prone to be affected by the patients’ general condition including fatigue, pain, and post-stroke depression, which makes it difficult for clinicians to determine the patients’ status^10^. This makes it difficult to evaluate the patients’ status objectively.

Electroencephalography (EEG) directly measures the cortical activity and reflects the brain’s spatio-temporal information. Compared to other brain imaging techniques it is relatively inexpensive, simple, and has almost no contraindications. Further, it has a high temporal resolution and provides electrophysiological information that other imaging modalities or clinical examinations do not^11^. Specifically, EEG enables physicians to evaluate the brain in real-time and quantify brain function objectively regardless of the patient’s cooperation. Therefore, there is widespread use of EEG in the neuroimaging field due to the ever-increasing interest in the temporal dynamics of the brain networks^12^. Moreover, EEG reflects the extracellular currents mainly from the dendrites of cortical pyramidal cells and has been reported to be sensitive enough for detecting cerebral ischaemic change^13^. Consequently, numerous EEG studies have been conducted over the past decades which have focused on evaluating brain function and identifying the EEG biomarkers related to brain injury and recovery. Sainio et al. reported that a high proportion of delta and a low proportion of alpha power were potentially strong indicators of poor outcomes after an ischaemic stroke^14^. Similarly, delta and alpha activities reportedly correlate with the degree of aphasia in subacute stroke^15^. Moreover, several reports indicate that quantified EEG based on power spectrum analysis allows stroke severity categorisation and clinical outcome prediction^16–20^. Furthermore, brain connectivity evaluation through quantification of the between-hemisphere spectral asymmetry was found not only to correlate with the NIHSS but also provide information regarding brain reorganization^21^.

Secondary stroke prevention is one of the most important issues in the subacute stroke phase since haemorrhagic transformation or reinfarction can deteriorate the prognosis^22^. Therefore, sequential evaluation of the neurological status and early detection of the stroke progression are paramount during admission. However, it is difficult to repeat neuroimaging due to both cost and accessibility. Additionally, clinicians cannot monitor the brain in real-time, which complicates the situation as clinicians usually depend on the clinical symptoms. In contrast, EEG provides dynamic cortical activity in real-time and has been explored for its potential utility in monitoring ischaemic brain injury^23,24^. Therefore, EEG is widely used as an intraoperative monitoring tool if there is a risk of perioperative cerebral ischaemia such as during a carotid endarterectomy^25,26^. However, in stroke, few studies have attempted to monitor the brain directly^27,28^, and there are not enough studies which evaluate the correlations of the brain’s anatomical injury with EEG results, since the majority of studies used EEG to only predict the clinical outcomes^13,29,30^.

Strokes can affect any part of the cerebral cortex. Among them, temporal lobe infarction is a common stroke subtype with various sequelae such as post-stroke language disorders and auditory dysfunction^31–33^. Despite its importance in processing auditory information, there are few basic research studies which assess temporal lobe infarction; this could be attributed to the difficulty in developing experimental models and evaluating the post-stroke neurological changes. Consequently, there is a lack of research on brain alterations, including electrophysiological changes, after a temporal lobe infarction. Furthermore, little is known about the auditory evoked potentials (AEPs) in stroke, while various other evoked potentials such as visual evoked potentials, somatosensory evoked potentials, and motor evoked potentials have been used to evaluate the brain state after a stroke^34^.

In this study, we aimed to investigate EEG characteristics after temporal lobe infarction involving the auditory cortex. Moreover, we aimed to identify EEG biomarkers related to ischaemic brain injury. A previous study reported that the right primary auditory cortex (A1) cortex of rats, which is part of the temporal lobe, plays a dominant role in the recognition of frequency-modulated sound^35^. Therefore, we expected that changes in cerebral neural activity in response to specific sounds could be measured using EEG after auditory cortical infarction. It is known that the biological timescales of post-stroke recovery are different between rats and humans^36^. Since active rehabilitation treatment and monitoring occurs during the subacute stroke phase, we assessed the rat EEG at 72 h post-stroke, which could be regarded as the subacute stroke phase in rat^37^. Specifically, we conducted a power spectral density (PSD) analysis and measured the peak amplitudes and latencies of AEPs in response to sound stimuli. Finally, we proposed a multiple linear regression model for quantifying stroke lesion volume using stroke-related EEG biomarkers.

## Results

### Analysis of EEG data and ischaemic lesion volume

Table 1 summarises the experimental results for each group. First, the results of the Kruskal–Wallis test indicated between-group differences in the stroke lesion volume (*p* < 0.001). The higher laser intensity caused larger ischaemic lesions and thus suggests that the different degrees of structural brain damage is concomitant with the laser intensity across the groups.

**Table 1:** Characteristics of EEG parameters and stroke lesion volume for the control group (n = 10), mild stroke group (n = 7), moderate stroke group (n = 7), and severe stroke group (n = 7).

Variables	Control	Mild Stroke	Moderate Stroke	Severe Stroke	Test statistic	*p* value
**Without auditory stimulation**						
RP beta	0.08 ± 0.09	0.09 ± 0.03	0.10 ± 0.04	0.10 ± 0.04	2.059 (3)	0.560
RP alpha	0.08 ± 0.05	0.07 ± 0.06	0.08 ± 0.04	0.07 ± 0.03	1.828 (3)	0.609
RP theta	0.19 ± 0.02	0.18 ± 0.02	0.18 ± 0.02	0.19 ± 0.02	4.022 (3)	0.259
RP delta	0.63 ± 0.12	0.66 ± 0.08	0.65 ± 0.05	0.63 ± 0.07	0.228 (3)	0.973
DAR	7.74 ± 7.31	9.30 ± 8.33	8.35 ± 5.66	9.26 ± 7.41	1.738 (3)	0.628
DTABR	4.64 ± 5.43	5.98 ± 3.03	4.57 ± 2.73	4.88 ± 3.30	0.494 (3)	0.920
**With auditory stimulation**						
RP beta	0.17 ± 0.07	0.11 ± 0.04	0.10 ± 0.01	0.12 ± 0.04	6.967 (3)	0.073
RP alpha	0.14 ± 0.02	0.10 ± 0.03	0.09 ± 0.01	0.09 ± 0.02	22.388 (3)	0.001^a,b,c^
RP theta	0.31 ± 0.06	0.29 ± 0.04	0.25 ± 0.03	0.23 ± 0.03	19.427 (3)	< 0.001^b,c^
RP delta	0.40 ± 0.07	0.49 ± 0.03	0.56 ± 0.02	0.58 ± 0.04	25.951 (3)	< 0.001 ^a,b,c^
DAR	3.01 ± 0.91	4.78 ± 1.50	5.89 ± 0.72	6.27 ± 2.29	24.566 (3)	< 0.001 ^a,b,c^
DTABR	2.18 ± 1.03	3.46 ± 1.19	4.02 ± 0.51	3.70 ± 2.02	12.600 (3)	0.006^b,c^
Amplitude (μV)	137.19 ± 50.14	124.16 ± 30.86	71.40 ± 32.49	51.18 ± 12.93	14.829 (3)	0.002^c^
Latency (s)	0.16 ± 0.03	0.20 ± 0.03	0.25 ± 0.02	0.27 ± 0.03	25.878 (3)	< 0.001^a,b,c^
Lesion volume (mm^3^)	-	3.33 ± 0.37	4.14 ± 0.11	4.65 ± 0.29	17.818 (2)	< 0.001†

Data are presented as the median ± interquartile range. The values of test statistics are expressed as chi-square value and (df). The *p* values were derived using a Kruskal–Wallis test.

^a^Represents statistical significance after Bonferroni correction between the control group and mild stroke group.

^b^Represents statistical significance after Bonferroni correction between the control group and moderate stroke group.

^c^Represents statistical significance after Bonferroni correction between the control group and severe stroke group. In addition, stroke lesion volume was significantly different between each group†

Next, we compared each EEG parameter in the stroke groups to those in the control group to identify parameters associated with post-stroke ischaemic injury. We conducted a PSD analysis in two different conditions; with and without auditory stimulation. In the absence of auditory stimulation, there were no significant differences in the relative power for each frequency band between the control and stroke groups. In contrast, in the auditory stimulation condition we found significant changes in the relative power for all frequency bands except for beta, the delta/alpha ratio (DAR), and the (delta + theta)/(alpha + beta) ratio (DTABR). More specifically, relative theta power in moderate and severe stroke group were smaller than that of the control group and DTABR in moderate and severe stroke group were larger than that of the control group. Furthermore, relative power in the alpha of each stroke group was smaller than that of the control group, whereas relative power in delta and DAR of each stroke group were larger than that of the control group.

**Figure 1:**
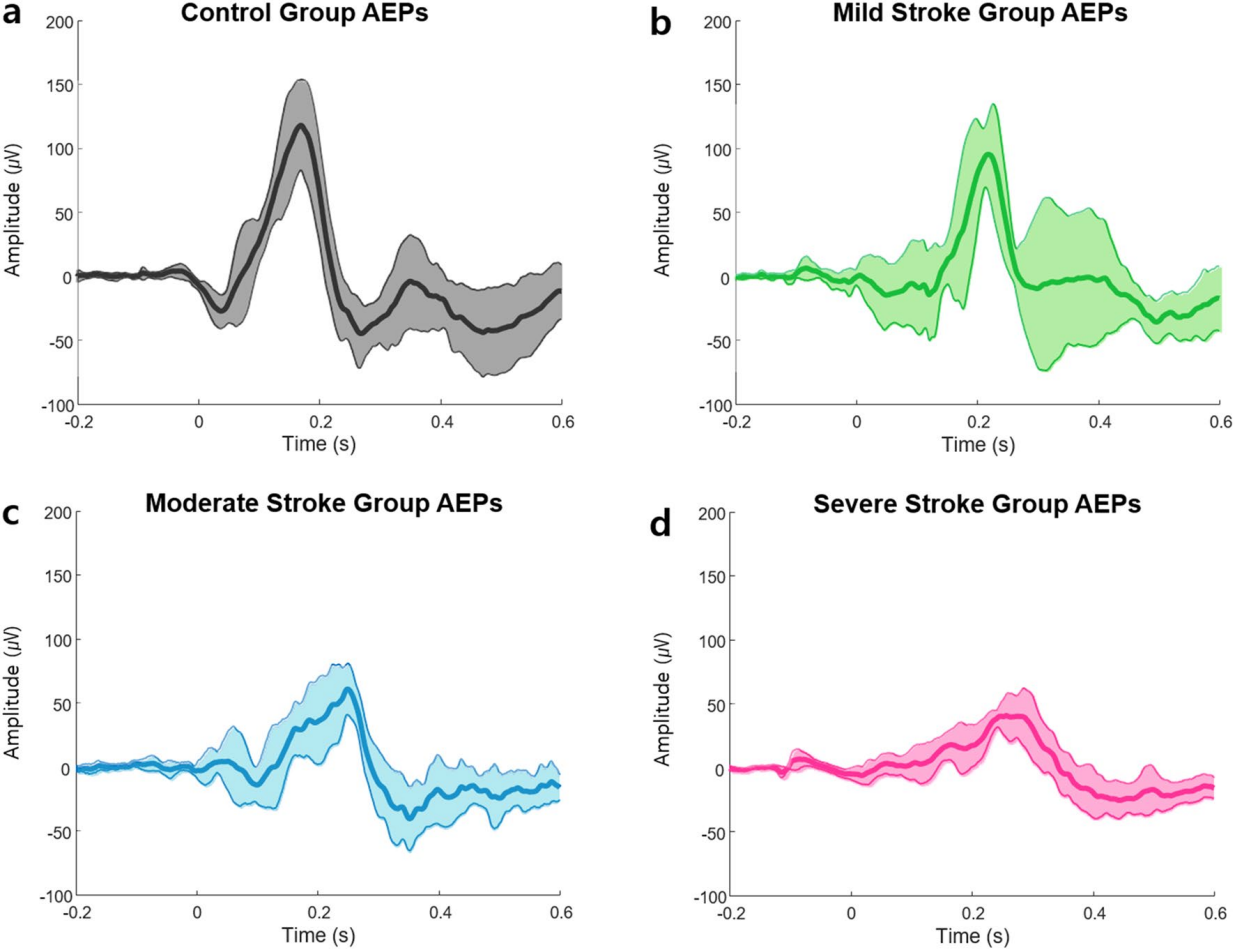
Mean AEPs waveforms of each group. (**a**) Control group, (**b**) Mild stroke group, (**c**) Moderate stroke group, and (**d**) Severe stroke group. The bold lines indicate the mean values, and the shaded bands represent the standard deviations. Note that the peak amplitudes became smaller, and the peak latencies were prolonged as strokes became more severe. AEPs, auditory evoked potentials.

In the AEPs analysis, we found that the amplitudes decreased as the stroke severity increased. Accordingly, the amplitudes were significantly smaller in the severe stroke group than that in the control group. Moreover, prolonged latencies were observed with increased ischaemic brain injury severity. Consequently, the latency in the mild stroke group was significantly prolonged compared to that in the control group. Figure 1 presents the mean AEP waveforms in each group. Supplementary Figure S1 shows the individual waveforms, and Supplementary Figure S2 demonstrated the four averaged AEP waveforms on a single graph, which highlights the intergroup differences.

These findings indicate that the relative powers in the alpha, theta, and delta, DAR, and DTABR as well as the amplitude and latency of the AEPs under auditory stimuli are the EEG parameters that reflect post-stroke brain injury.

### Correlations between EEG parameters and stroke lesion volume

**Figure 2:**
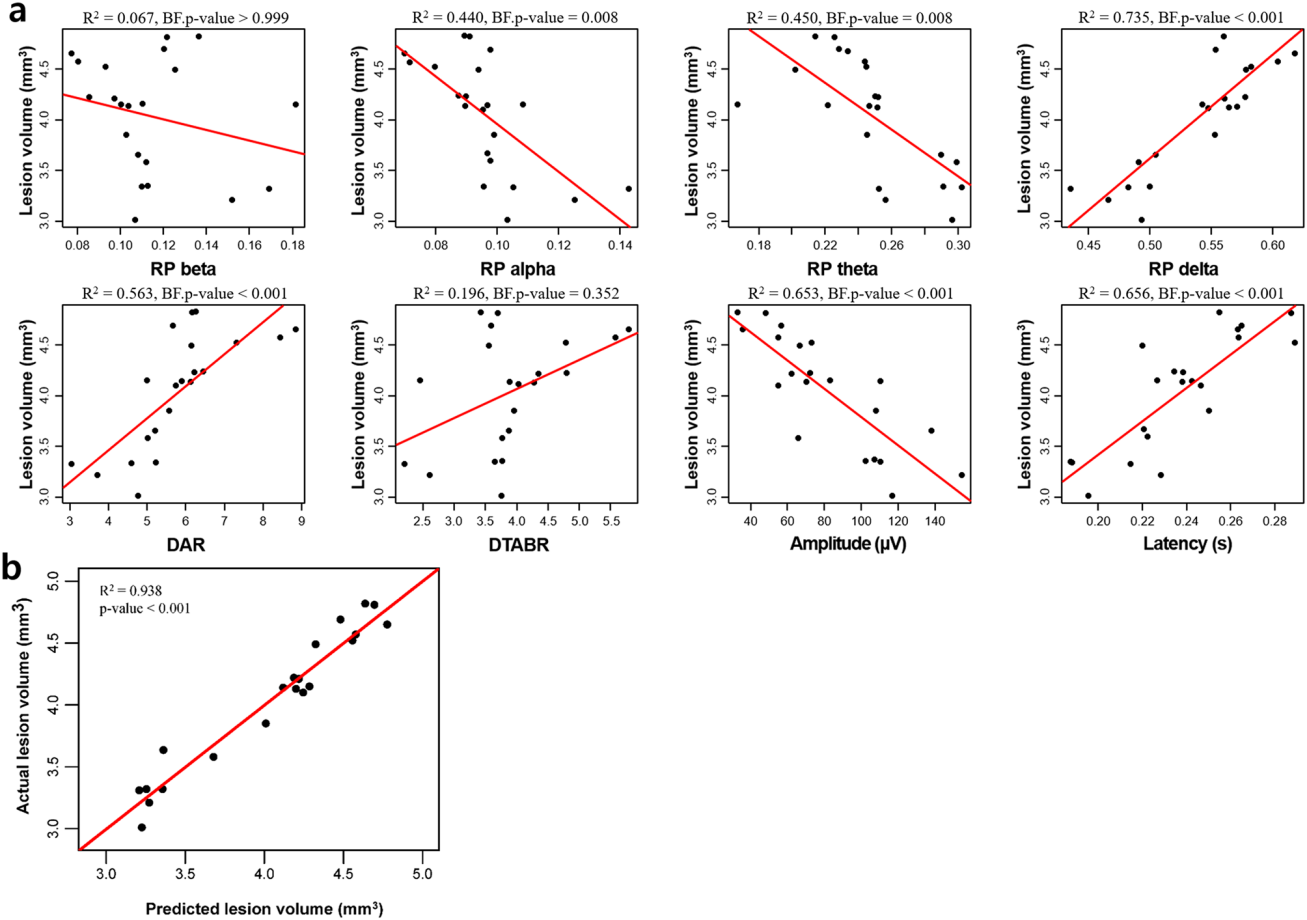
Scatter plots of individual data. (**a**) Scatter plots showing the relationship between each stroke-related EEG parameter and stroke lesion volume and (**b**) predicted stroke lesion volume using multiple linear regression model and actual stroke lesion volume. DAR, delta/alpha ratio; DTABR, (delta + theta)/(alpha + beta) ratio. BF.*p* value indicates Bonferroni corrected *p* values.

Next, we selected EEG parameters that demonstrated significant differences between the control and stroke groups (Table 2). We analysed the correlations between the EEG parameters (relative powers for alpha, theta and delta, DAR, DTABR, and amplitude and latency of AEPs) and the stroke lesion volume. We only included data from the stroke groups as this study investigated the feasibility of EEG for monitoring ischaemic lesion volume after a stroke. We conducted a linear regression analysis to identify EEG parameters that could quantify brain damage. We found a high correlation between all of the selected EEG parameters, except for DTABR, with stroke lesion volume (Table 2). There were positive correlations between the relative delta power, DAR and latency with the stroke lesion volume. In contrast, there were negative correlations between the relative powers in the alpha and theta, and amplitude with the stroke lesion volume (Bonferroni corrected *p* < 0.05). Relative delta power had the highest correlation with the stroke volume (β = 0.857). The R^2^ of relative delta power was 0.735, which is the proportion of variance in the dependent variable explained by the independent variable. Individual correlations between each stroke-related EEG parameter and stroke lesion volume are presented as scatterplots (Fig. 2a).

**Table 2:** Univariate linear regression analysis for stroke lesion volume.

EEG parameters	Unstandardized coefficients		Standardized coefficients	R^2^	BF.*p* value
	B	SE	Beta		
RP alpha	− 23.541	6.097	− 0.663	0.440	0.008
RP theta	− 11.519	2.764	− 0.691	0.450	0.008
RP delta	10.248	1.411	0.857	0.735	< 0.001
DAR	0.316	0.064	0.751	0.563	< 0.001
DTABR	0.286	0.133	0.443	0.196	0.352
Amplitude	− 0.014	0.002	− 0.818	0.653	< 0.001
Latency	16.432	2.624	0.821	0.656	< 0.001

BF.*p* value indicates Bonferroni corrected *p* values. The BF.*p* values were calculated by multiplying *p* values by the number of the tests using R software^75^. The residual errors for each model showed normal distribution on a Shapiro–Wilk test (*p* value > 0.05).

### Multiple analyses using EEG parameter combinations for stroke lesion volume estimation

**Figure 3:**
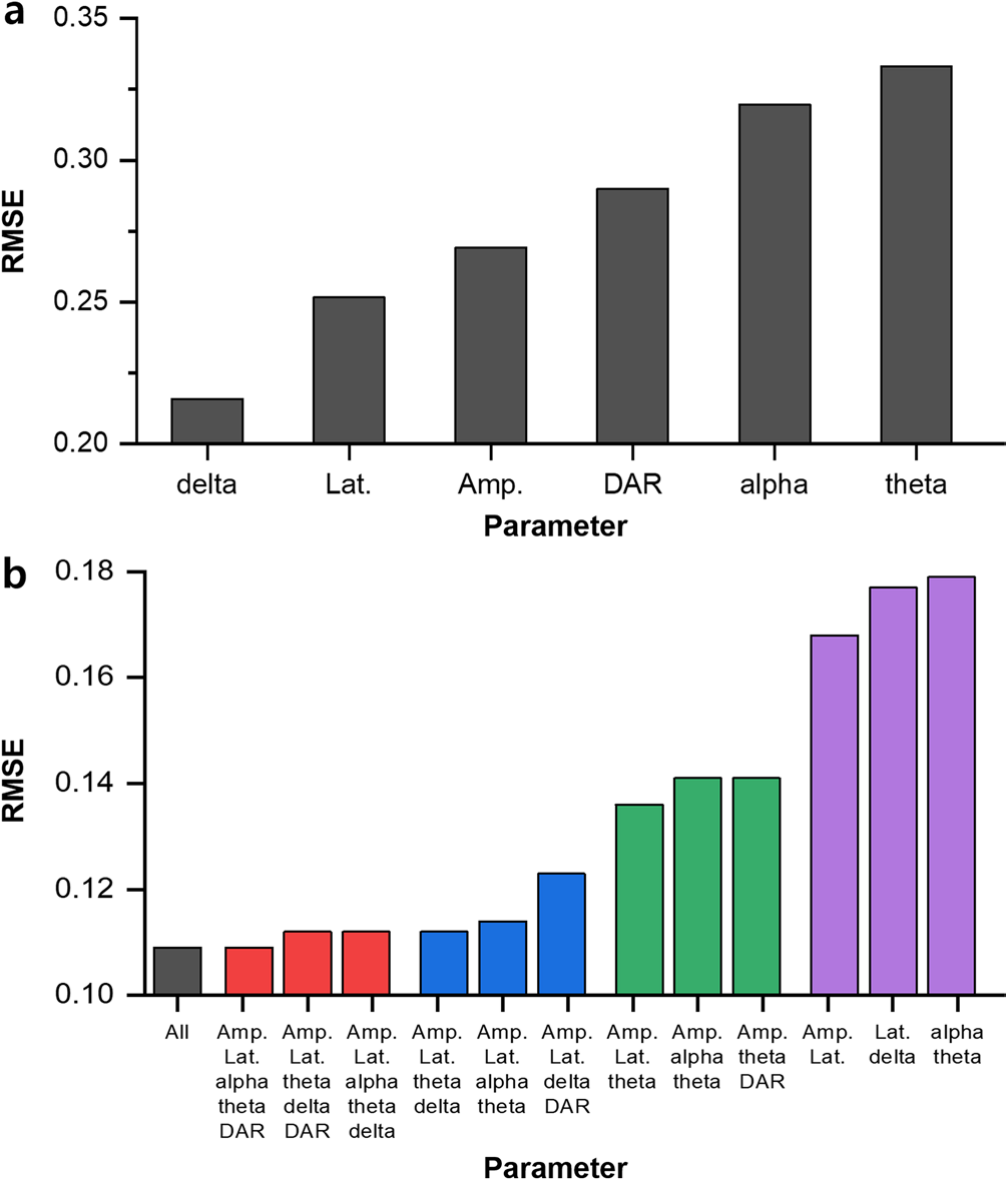
RMSE values for each predictive model. (**a**) RMSEs of the univariate linear regression model. The model using the relative delta power parameter most accurately predicted the stroke lesion volume (RMSE = 0.216) followed by the model using the latency (RMSE = 0.252). (b) RMSEs of the multiple linear regression model. RMSEs with every possible combination of the EEG parameters were calculated. The best three RMSE results based on each number of input features are shown. A trend of increasing accuracy was observed as the number of EEG parameters increased. As a result, the regression model using all the selected EEG parameters exhibited the highest accuracy (RMSE = 0.109). DAR, delta/alpha ratio; RMSE, root mean square error.

We calculated the root mean square error (RMSE) value to evaluate the accuracy of the predictive models with the selected EEG parameters in Table 2. In this study, we defined RMSE as the standard deviation of the residuals between the observed and estimated stroke lesion volume. First, we calculated the RMSE for the univariate linear regression model. Figure 3a presents the RMSEs for each EEG parameter which demonstrated significant correlations with the stroke lesion volume. Among them, the univariate linear regression model using relative delta power was the best-fitting regression model (RMSE = 0.216) while the regression model using relative theta power was the least accurate for predicting the lesion volume (RMSE = 0.333).

Next, we calculated the RMSEs for the multiple linear regression models with every possible combination of *n* EEG parameters. Specifically, we used every parameter combination used as an input feature for linear regression, which yielded 63 RMSE values (2^6^ − 1 = _6 (number of all selected features through univariate linear regression)_ C_6 (number of input features for regression)_ + _6_C_5_ + _6_C_4_ + _6_C_3_ + _6_C_2_ + _6_C_1_). Figure 3b presents the RMSEs for the regression models using all six selected EEG parameters and the three best RMSE results in each group based on the number of input features. As expected, the regression model using all of the selected EEG parameters demonstrated the highest accuracy (RMSE = 0.109). Furthermore, we calculated the Akaike information criterion (AIC) and Bayesian information criterion (BIC) for each model (for more detailed information see Supplementary Table S1). The regression model using four EEG parameters (relative powers in the theta and delta, amplitude, and latency of AEPs) exhibited the lowest AIC and BIC values, likely due to these statistical methods introducing a penalty term for the number of parameters in the model.

### A predictive model for quantifying brain injury using the EEG parameters

Finally, we developed a multiple linear regression model for quantifying the stroke lesion volume. We used stepwise regression algorithms to select the appropriate subset of explanatory variables. Additionally, we conducted multicollinearity diagnostics as some of the EEG parameters were highly correlated (Supplementary Figure S3). Table 3 presents the results of the stepwise linear regression analysis. As a result, we derived the following equation for the final model:

$$Predicted\, stroke\, lesion\, volume = \left( { - 3.962} \right) \times relative\, theta\, power + 3.217 \times relative\, delta\, power + 6.186 \times Latency + \left( { - 0.006} \right) \times Amplitude + 2.312$$

**Table 3:** Stepwise linear regression analysis using optimal EEG parameters for stroke lesion volume.

EEG parameters	Standardized coefficients	Standardized coefficients	Standardized coefficients	*p* value	Collinearity statistics	
	B	SE	Beta		Tolerance	VIF
**Step 1**						
(Constant)	− 1.501	0.766		0.065		
RP delta	10.248	1.411	0.857	< 0.001	1.000	1.000
**Step 2**						
(Constant)	− 1.633	0.620		0.017		
RP delta	6.664	1.574	0.556	0.001	0.526	1.902
Latency	8.768	2.636	0.438	0.004	0.526	1.902
**Step 3**						
(Constant)	0.501	0.815		0.547		
RP delta	4.240	1.457	0.355	0.010	0.395	2.531
Latency	7.375	2.157	0.368	0.003	0.506	1.978
Amplitude	− 0.006	0.002	− 0.359	0.004	0.499	2.003
**Step 4**						
(Constant)	2.312	0.882		0.019		
RP theta	− 3.962	1.274	− 0.238	0.007	0.665	1.504
RP delta	3.217	1.230	0.269	0.019	0.367	2.726
Latency	6.186	1.797	0.309	0.003	0.483	2.072
Amplitude	− 0.006	0.002	− 0.353	0.001	0.449	2.004

R^2^ = 0.735 for step 1, R^2^ = 0.836 for step 2, R^2^ = 0.900 for step 3 and R^2^ = 0.938 for step 4 with *p* value < 0.001. Collinearity statistics of variables excluded from the final model are as follows: tolerance = 0.063, VIF = 15.943 for RP alpha and tolerance = 0.067, VIF = 14.870 for DAR. The residual errors of the final model showed normal distribution on a Shapiro–Wilk test (*p* value = 0.648). VIF indicates variance inflation factor.

The analysis indicated that the predictive model had a statistically significant high explanatory power (R^2^ = 0.938, *p* < 0.001). There was a positive correlation between the relative delta power and latency with the stroke lesion volume. In contrast, there was a negative correlation between the relative theta power and amplitude with the stroke lesion volume. Relative alpha power and DAR were excluded from the final model as they demonstrated multicollinearity. Individual levels of the predicted stroke lesion volume against the stroke lesion volume are plotted in Fig. 2b.

We further analysed the stroke groups’ data to determine whether we could predict stroke lesion volume using unseen EEG data. To overcome the limited sample sizes, we separated the original stroke dataset randomly into the training (n = 18) and test sets (n = 3) and constructed a multiple linear regression model using only the training set. Then we calculated the RMSE value with the test set and iterated the process a 1000 times to avoid selection bias. As a result, we obtained 1000 RMSE values and the frequencies of the selected EEG parameters for each model (Supplementary Figure S4). Based on these results, we can assume that we can quantify the stroke lesion volume with unseen data and that the EEG parameters which were selected in the final linear regression models (relative powers in theta and delta and latency and amplitude of AEPs) are truly important variables.

## Discussion

Previous studies indicate that comprehensive monitoring and assessment of brain could provide valuable insights into the neural basis of stroke recovery^38–40^. Generally, neuroimaging techniques such as MRI or PET are preferred in acute stroke care settings. However, their accessibility and cost make it difficult to implement them for repetitive monitoring^27^. Consequently, EEG has emerged as an alternative method for post-stroke brain monitoring in both clinical and research settings^24,30,37,41,42^. In this study, we evaluated the correlations between neural activity and brain structural damage in rats after focal cerebral ischaemia in the right auditory cortex. We hypothesised that strict control of the confounding factors and accurate epidural EEG recording through basic animal experiments could address the current shortcomings of clinical research. Further, it allows for clearer identification of post-stroke neural activity characteristics in a situation where previous studies are insufficient. In the linear regression analysis, we observed a strong correlation between relative powers in the alpha, theta and delta, and DAR, as well as the amplitude and latency of AEPs with the stroke lesion volume. Furthermore, we developed a multiple linear regression model with a high explanatory power that could quantify stroke lesion volume through epidural EEG signals from a single channel. These findings highlight the feasibility of utilising EEG and the observed stroke-related EEG features for stroke monitoring which have rarely studied before. To the best of our knowledge, this is the first study to use the auditory cortical stroke model and predict structural ischaemic damage using epidural EEG in rats.

Over the past two decades, EEG has progressed from conventional EEG scoring to quantitative EEG analysis, such as the PSD analysis applied in the present study^23^. The standard PSD analysis based on the canonically pre-defined frequency bands has been recently criticised since it did not consider the aperiodic components such as the 1/f signals^43,44^. However, several other studies have reported a strong correlation between the quantified changes in fast and slow frequency bands with clinical outcomes and post-stroke injury severity. Although most of the studies analysed the resting-state EEG, there is growing evidence of a strong correlation between the increased delta power^13^ and the decreased alpha and beta rhythms^16,45^ in the ischaemic hemisphere with stroke lesion location and poor clinical outcomes. Further, theta activity has been reported to correlate with cognitive impairment^46^ and reduced language processing^47^ and could be used to discriminate patients with stroke from healthy controls^48^. Moreover, post-stroke enhancement of theta activity has been reported^30^. Further, Finnigan et al. reported that the DAR was the most optimal classifier followed by DTABR and relative power in delta for discriminating patients with acute ischaemic stroke from the healthy controls^20^. A recent study recorded the EEG data from patients with stroke using a wireless single-channel EEG system and demonstrated the prognostic value of DAR and the delta/theta ratio (DTR) as potential markers of post-stroke cognitive function^30^.

Moreover, several studies exist on the correlations between the pathophysiological state of the brain and post-stroke EEG changes. An observational study reported a correlation of increased delta activity and decreased alpha activity with cerebral blood flow in cerebral ischaemia^49^. Moreover, delta activity has been reported to be associated with post-stroke oedematous changes in distal dendrites^50^ while alpha activity demonstrates the integrity of synaptic networks of the cortex^51^. Consistent with these previous findings, we observed an increased relative delta power and a decreased relative alpha power in the stroke groups, with this tendency becoming more evident with stroke severity. The beta activity is indicative of neuronal survival. However, it is often considered to be an unreliable index of post-stroke pathophysiology since it is prone to be affected by artefacts, particularly those at higher frequencies^16^. Similarly, there was no significant difference in the relative power in beta between the control and stroke groups in this study. Although increased theta activity is known to be related to hypoperfusion, it has been criticised as being an insignificant marker for post-stroke monitoring given that slow alpha activity is a confounding factor for its relative power^20^. Further, previous animal studies have reported inconsistent results; specifically, post-stroke increases^37^ and decreases^52^ the theta wave activity. In the current study, we found a significant negative correlation of relative theta power with the stroke lesion volume. However, we should be careful not to interpret any single EEG parameter to be particularly meaningful in the stroke condition, since they all seem to be highly correlated to each other as shown in Supplementary Figure S3, and thus high collinearity exists in the final model.

**Figure 4:**
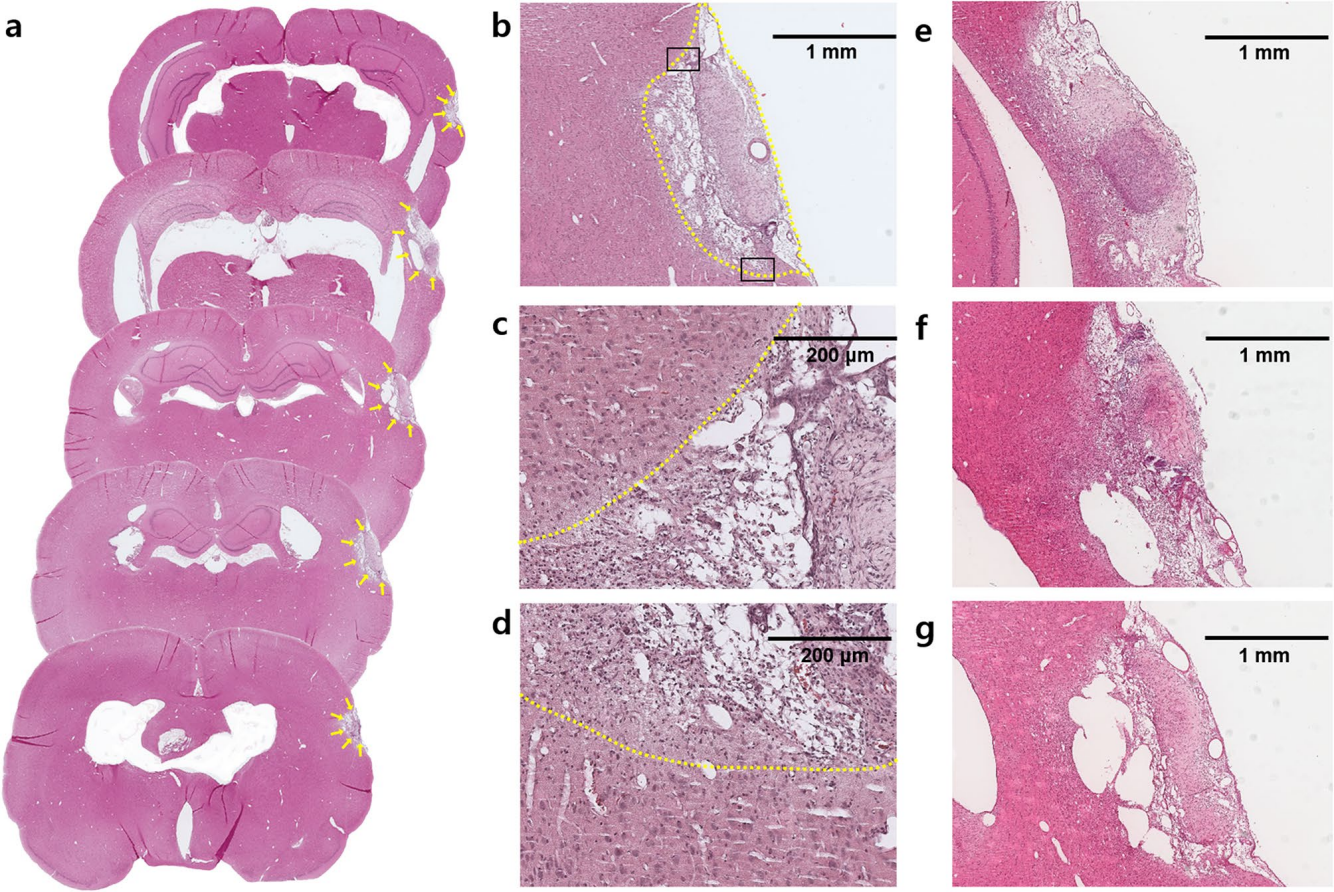
Histologic findings of stroke models on haematoxylin and eosin staining. (**a**) An overview of multiple coronal sections of the rat brain showing the stroke lesion from rostral (top) to caudal (bottom). The arrows indicate the boundary of the ischemic lesion. (**b**) An example of the coronal section indicating the outline of the infarcted area shown by the dashed line. (**c**,**d**) High-power view of the boxed regions in (**b**). The demarcated zone of pale-staining cortex indicates the infarcted area, surrounded by mononuclear inflammatory cell infiltration. (**e**) A coronal section of mild stroke, (f) moderate stroke, and (**g**) severe stroke. As the stroke became severe, destructive changes such as cystic cavitation also increased.

AEPs are electric potentials generated by the synchronous firing of neighbouring neural populations in the brain, which are time-locked responses to the specific acoustic stimulation^53^. Small-voltage signal deflections are considered to reflect physiological changes in the auditory pathway and the electrical potentials have been reported to have great sensitivity in revealing the functional integrity of the brain^54^. AEPs are widely used in clinical practice given their high temporal resolution and their ability to determine neural generators of the electrical responses. Previous stroke studies suggest that AEPs directly reflect the functional state of the brain and can be used for neurophysiologic assessment, including prognosis prediction^55^, evaluation of brain reorganization^56^, and post-stroke language function^57,58^. Oddball paradigm is a commonly used task for evaluating the cognitive function in event-related potential studies^59,60^. In contrast, brainstem AEPs are recorded under the same repetitive clicks that evaluate the brain stem auditory pathway^34^. However, little is known about the AEPs under the consistent auditory stimuli after a stroke. We assumed that the extent of auditory cortical injury could be accurately evaluated by the evoked potentials since they are generated by cortical pyramidal cells^61^. Therefore, we acquired AEPs by summing the EEG signals for the specific sound stimuli and analysed the AEP profiles in terms of amplitude and latency based on the stroke severity. Generally, the amplitude and latency of AEPs reflect the temporal integration of cerebral neural activity^62^. More specifically, decreased amplitudes reflect the decreased neural activation resulting from axonal degeneration and neural transaction while prolonged latencies indicate diffuse demyelination and conduction block of the neural circuit^63^. We did not find significant amplitude differences in the mild and moderate stroke groups when compared to that in the control group. We only observed a significant decrease in the amplitudes in the severe stroke group compared to that in the control group. Moreover, there was prolonged latency in each stroke group, that was positively associated with stroke severity, and was significantly different than that of the control group. These changes in AEPs suggest that the aetiology of mild and moderate strokes in this study was mainly the diffuse demyelination. On the other hand, the aetiology of severe stroke was not only demyelination but also severe neural injury resulting in axonal damage and neuronal fibre loss. These findings are consistent with the histologic findings presented in Fig. 4. We observed more structural integrity loss with liquefactive necrosis with increased stroke severity. These findings demonstrate that the amplitude and latency of AEPs accurately reflect the neuronal brain states and are appropriate predictors for estimating post-stroke structural brain damage.

The main requirement for EEG signal interpretation is good signal quality. In this respect, animal studies enhance the investigation of pathophysiologic EEG features since they allow for the acquisition of more accurate signals through invasive procedures such as epidural electrode implantation. Therefore, we expected that regression modelling using EEG in animal experiments could accurately reflect the extent of ischaemic brain injury. Given the complexity of the auditory signal processing mechanism in the brain, the multiple linear regression model using the EEG parameter combination strengthened the explanatory power compared to the univariate linear regression models (Fig. 3). Although there are insufficient animal studies, recent clinical studies on the post-stroke brain state have developed regression models using EEG. Wu et al. analysed the correlation between infarct volume and EEG and utilised the ipsilesional and contralesional delta power to predict the infarct volume^27^. Further, Aminov et al. developed a regression model combining DTR with other clinical ratings of neurological status that explained 75% of the variance in post-stroke cognitive function^30^. Moreover, recent studies have used a single saline net^27^ or wireless single-channel electrode systems^30^ rather than the traditional applications of multiple separate electrodes to improve usability and portability. Accordingly, we recorded the cortical activity using a single epidural EEG. This allowed us to present a regression model with a high explanatory power that could quantify the extent of brain injury with a small RMSE. However, we should keep in mind that this study analysed the EEG data obtained under anaesthesia and part of the results could be affected by the anaesthetic effects. Slowing of the frequency with an increase in delta power is a typical finding of EEG changes after isoflurane anaesthesia^64^. Further studies are required in rats in an awake state.

Resting-state EEG might be insufficient for recording subtle post-stroke changes^65^. Therefore, there is a need for studies recording EEG under certain tasks or stimuli to further examine the cerebrovascular diseases, given EEGs superior sensitivity over resting-state conditions^28^. In this study, both EEG signals with and without the auditory stimuli were analysed. Interestingly, no significant EEG differences were observed between each group when there was no auditory stimulation. Overall, relative delta power was dominant across all the groups when compared to the results under the auditory stimulation. On the other hand, subtle changes of EEG became more pronounced under the auditory stimulation and allowed for the identification of stroke-related EEG features. The high R^2^ value obtained in this study could be attributed to more accurate signal acquisition with implanted electrodes as well as the fact that the severity of ischaemic brain injury could be accurately reflected on EEG under the passive stimulation paradigm. However, this does not conclusively determine that the combinations of predictors used in the regression model were the optimal EEG parameters. The model might not fit well with changes in the subjects’ characteristics or stroke types since the sample size did not allow for generalizability. Additionally, there is a possibility that the changes in relative power of each frequency band are mainly affected by the changes in AEPs. However, this study provides neurobiological insight that proper combinations of EEG signals could accurately reflect post-stroke structural brain damage.

In this study, we investigated the stroke-related EEG biomarkers and provide evidence that EEG could successfully estimate the structural stroke severity. Further, we used the temporal lobe infarction model, a common stroke type that has been rarely studied in animals. Although this is an early-stage animal model, we believe that it could be improved and may prove crucial for studies on temporal lobe infarction and related disabilities such as aphasia. However, further studies are required to increase the value and robustness of our findings. We could solely focus on the effects of stroke on EEG by minimising the variability such as demographic features and strictly controlling the pathophysiological conditions^66^. However, studies with different stroke subtypes and phenotypes including female rats with varying age are required to increase the generalizability of the predictive model. Additionally, analyses of EEG under different duration and frequency sounds are required since the duration of the sound stimuli in this study was relatively long and there would have been a constant sound input to the brain, which may have generated a range of overlapping EEG activity. Explicit parameterisation of neural power spectra to specify periodic and aperiodic activity would help obtain more accurate EEG parameters. Moreover, it is necessary to analyse the effects of anaesthesia on EEG. Finally, there is a need for more advanced studies, including clinical studies, to allow for the successful clinical application of EEG for stroke monitoring since translational animal studies have inevitable limitations regarding their practical application.

In conclusion, accurate monitoring and determining the extent of brain injury are crucial for stroke-related care. We investigated the feasibility of EEG for evaluating post-stroke neural activity. We acquired EEG signals using a single epidural electrode and analysed the correlations between EEG parameters and the extent of ischaemic brain damage in a rat model of focal cerebral ischaemia. Regression analysis indicated that the relative powers in the alpha, theta and delta, DAR, and DTABR as well as the amplitude and latency of AEPs, could be considered as electrophysiological biomarkers for post-stroke brain injury, particularly for a temporal lobe stroke involving the auditory cortex. Further, combinations of EEG predictors (relative powers in the theta and delta, and AEP latency and amplitude) produced a powerful model for quantifying post-stroke structural brain damage. Since this is a pre-clinical animal study, there is a need for further studies to generalise and optimise the model. However, this study provides evidence that EEG is useful as a supplemental neurophysiological assessment method.

## Methods

### Animals

The total sample size required was calculated as 10 subjects for the control group and 25 subjects for the stroke groups, based on previous studies^28,30,37^ and 10% dropout rate from a pilot study. We only included male rats to minimise the potential confounders which could affect the study results, including effects of oestrogen. As a result, the study included 35 male Sprague–Dawley rats (337.42 ± 12.27 g, 10 weeks of age, Orient Bio Inc., Seongnam, Korea). Initially, the rats were randomly assigned to the mild stroke (n = 8), moderate stroke (n = 8), severe stroke (n = 9), and control groups (n = 10). Then, all the rats underwent the surgical procedure, and 33 rats survived the experiment. Two rats from the severe stroke group perished 1 to 2 days after the stroke induction. In addition, 1 rat from the mild stroke group and 1 rat from the moderate stroke group were excluded from the study as the EEG data from the subjects were extremely noisy during the signal acquisition. In summary, a total of 31 rats completed the experiment, and all of the EEG data and stroke lesion volume from these rats were included in the final analyses.

The rats were individually housed in plastic cages and maintained on a 12-h light/dark cycle at a temperature of 21 ± 1 °C with ad libitum access to food and water. All of the experimental animal procedures were conducted in compliance with the guidelines of the Institutional Animal Care and Use Committee of the Gwangju Institute of Science and Technology (GIST). The study was approved by the Institutional Review Board of GIST (approval number: GIST-2019-047).

### Photothrombotic stroke model

The photothrombotic stroke model is commonly used in animal studies since it allows for the accurate induction of focal ischaemia in specific cortical regions. Although the photothrombotic stroke model does not induce the penumbra zone, which is one of the typical features of stroke, the model can precisely control the ischaemic lesion with high reproducibility. Before establishing the stroke model, we anaesthetised each rat using 5% isoflurane with oxygen gas (0.6 L/min flow rate) in an induction chamber. Once rats lost the righting reflex, they were removed from the induction chamber and an aesthetic nosecone was applied. The isoflurane gas mixed with oxygen was then redirected to the nosecone and reduced to an anaesthesia maintenance dose of 1.5%. Next, the rat was mounted onto a stereotactic frame and its head secured by inserting the ear bars into the ear canals. We shaved the fur from the ears to just in-between the eyes using a razor. A line block was performed with 2% lidocaine before incision. Subsequently, the skull was exposed by retracting each side of the scalp. Moreover, we partly removed the right temporalis muscle to expose the right auditory cortex. We then injected the Rose Bengal dye (30 mg/kg, Sigma-Aldrich, USA) into the tail vein. Next, we illuminated green laser light (532 nm, 4.0 mm beam diameter) over the right A1 cortex for 15 min to induce photothrombotic ischaemia^67^. The coordinates of the A1 cortex were 4 mm posterior, 7.6 mm lateral, and 4 mm ventral to bregma^68^. We adjusted the laser intensity power according to the experimental group. Previous studies have used a 17-mW laser output to create permanent photothrombotic stroke models and induce distinct structural brain damage in rats^67,69^. Therefore, we irradiated the rats with 17 mW, 11 mW, 5 mW green lasers and respectively designated them as the severe, moderate, and mild stroke groups, since laser intensity is known to be correlated with damage volume^70,71^. The sham-operated group underwent all of the aforementioned steps; however, the green laser was turned off during the 15 min exposure time. We maintained the rats’ core temperature at 37 °C during the surgery using an animal temperature controller (Temperature controller 69001, Scitech Korea, Korea).

### Electrode implantation surgery for EEG recording

**Figure 5:**
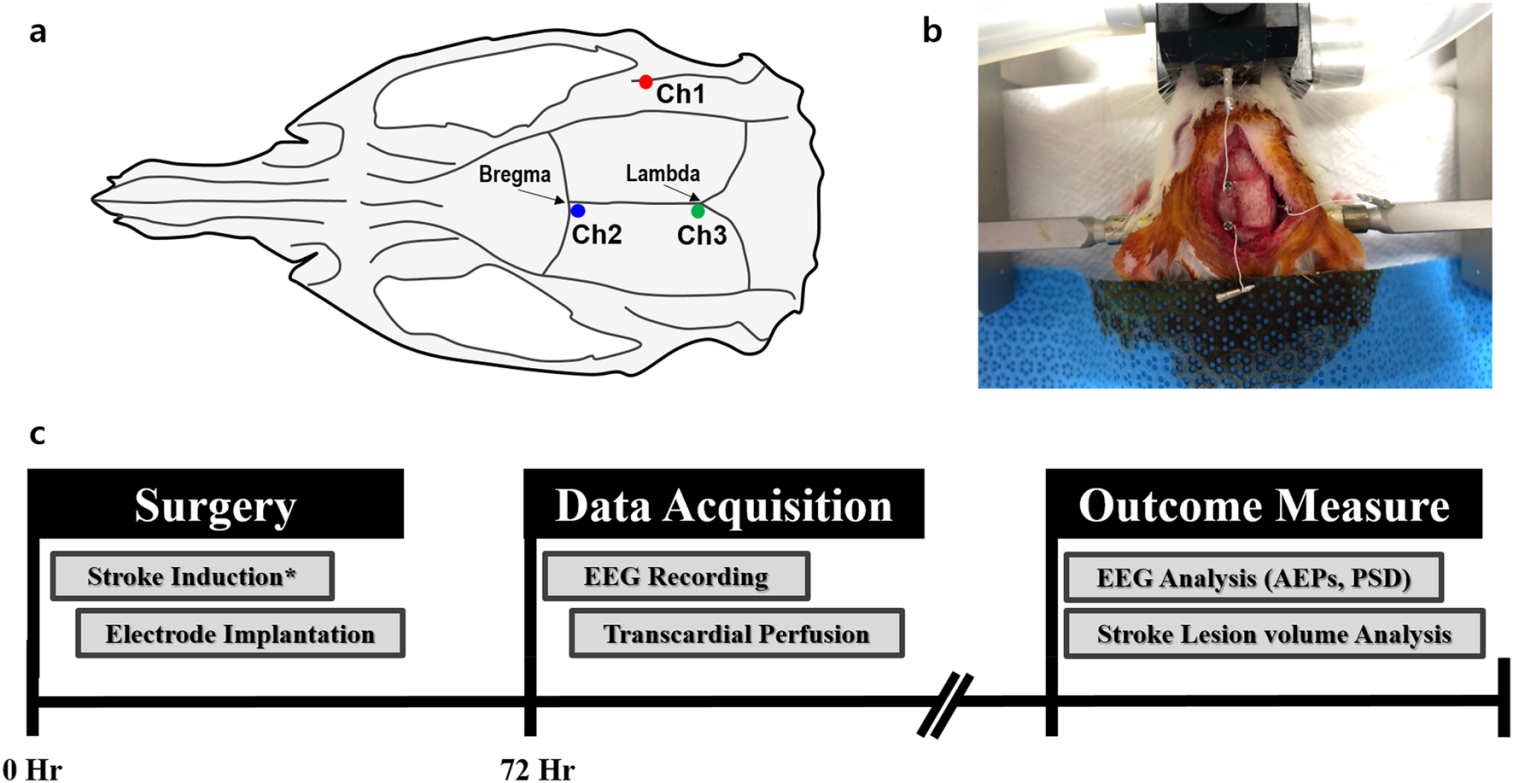
Schematic diagrams of the electrode placement for EEG recording and the experimental timeline. (**a**) Dorsal skull surface of rat showing locations of each electrode. Ch1 was located over right A1 cortex. Ch2 indicates reference electrode, while Ch3 indicates a ground electrode. (**b**) Electrodes were successfully implanted on the right A1 cortex, reference and ground. (The image was taken by H.-J.Y.) (**c**) Flowchart of the whole experiment. Photothrombotic stroke induction (only for each stroke group*) and microelectrode implantation surgery were performed under isoflurane anaesthesia. EEG signals were acquired from the right A1 cortex 72 h after the surgery. All the rats in the stroke groups were then transcardially perfused, and their brains were extracted to evaluate the stroke lesion volume. Finally, the EEG signals and stroke lesion volume were analysed offline.

Immediately after the stroke induction, all rats underwent the microelectrode implantation surgery to allow for the acquisition of EEG signals from the right auditory cortex. We performed a durotomy at 3 different sites with a dental drill to allow for EEG electrode placement. We drilled one hole over the right A1 cortex at the same location where the green laser was illuminated. Moreover, we drilled a hole for the reference electrode at 0.7 mm posterior and 0.8 mm left to bregma, and another for the ground electrode at 8 mm posterior and 0.8 mm left to bregma (Fig. 5a,b). After EEG electrode implantation, the electrodes were connected to a multi-pin connector. Finally, we covered the exposed skull with bone cement. We injected antibiotics (Ceftazol 20 mg/kg, Guju Pharma Co, Korea, IM) and an analgesic agent (Ketoprofen 2.5 mg/kg, Uni Biotech, Korea) for 3 consecutive days after the surgery.

### Sound stimulation and EEG acquisition

Studies have used frequency-modulated stimuli to investigate auditory perception in animals^35^. In this study, we used a frequency-modulated tone with a linearly increasing frequency from 8 to 12 kHz as a target sound, which covers the rat hearing range with a 750 ms duration for sound stimulation^72^. In addition, other three frequency-modulated stimuli (linearly increasing frequency from 4 to 8 kHz, and linearly decreasing frequency from 8 to 4 kHz and 12 to 8 kHz with a 750 ms duration) were used to minimise the habituation effects. Furthermore, 2 s of the silent period were randomly presented for 100 times between the sound stimuli to record EEG without auditory stimuli. All the sound stimuli were generated using the MATLAB software version 2017b (Mathworks, Inc., MA., USA). Therefore, the four types of frequency-modulated stimuli or the 2 s of the silent period were randomly presented for 100 times each with an inter-stimulus interval of 2 s. The total duration of EEG recording was around 1000 s.

We acquired EEG signal responses to sound stimuli from the right A1 cortex 72 h after surgery. We anaesthetised rats using 5% isoflurane in an induction chamber. Once rats lost the righting reflex, we applied 1.5% isoflurane via a nosecone during the recording time to prevent EEG signal contamination from motion artefacts. Next, we connected a multi-pin connector to a customised recording device (g.USBamp and g.HEADstage, g.tec medical engineering GmbH; Graz, Austria), which acquired signals at a 1200 Hz sampling frequency. We performed all recordings in a soundproof booth to maximise the signal to noise ratio.

### EEG signal processing

We analysed the EEG signals under the target sound offline using MATLAB. EEG signals without the auditory stimuli were also analysed. First, the recorded EEG data were band-pass filtered between 0.05 and 60 Hz. We used the zero-phase forward and reverse Infinite Impulse Response Butterworth filter of 4th order^73^. Further, we averaged the last 300 ms of the signal before stimulus onset as a baseline correction. Next, we down-sampled the data from 1200 to 600 Hz. Subsequently, we conducted the PSD analysis using signals obtained from the target stimulus or silent period onset to 1 s using Welch’s method, which is one of the most widely used periodogram methods for determining the power density of EEG frequency components^74^. The parameter was set to divide the EEG signals into eight sections of equal length, each with a 50% overlap based on the Hamming window. We defined the frequency range for each band as follows: Delta (1–4 Hz), theta (4–8 Hz), alpha (8–12 Hz), and beta (12–30 Hz). We calculated the relative power for each frequency band by summing all of the absolute PSD values across the four bands to compute the total power followed by dividing the absolute value for each frequency band with the total power. Finally, we calculated the DAR (delta/alpha ratio) and the DTABR ((delta + theta)/(alpha + beta) ratio), which were computed by the relative power of the relevant frequency bands.

Further, we analysed AEPs in response to the target sound stimuli by averaging all of the epochs between 300 ms before the stimuli onset to 500 ms after it. The AEP amplitude was defined as the highest recorded voltage following the sound stimulus. The latency of the components of the AEPs was defined as the duration from stimulus onset to the peak amplitude.

### Histological analysis of the stroke volume

All rats in the stroke group were transcardially perfused and fixed with formalin under deep anaesthesia on the same day after EEG signal acquisition. Brains were carefully extracted and fixed with 50 ml of 4% formalin for 24 h. After fixation, brains were placed in a 30% sucrose solution for 3–4 days, after which they were carefully removed and cryoprotected. Next, the brains were sliced coronally (thickness = 10 μm) using a cryostat machine and then mounted on microscope slides. Finally, the slides were counterstained with haematoxylin and eosin solutions (Fig. 4).

Two independent observers analysed the cortical stroke volume using the ImageJ software (NIH, Bethesda, MD, USA). The observers were blinded to the experimental condition. They manually traced the damaged ischaemic tissue in each section and calculated the stroke volume by multiplying its surface area by the slice thickness. Then, all the stroke volume for every slice were summed to determine the total stroke lesion volume^67^. A consensus was achieved after sufficient discussion between the observers if there were any differences in the analysis. The schematic of the experimental timeline is shown in Fig. 5c.

### Statistical analyses

We performed all statistical analyses using the SPSS software (SPSS version 20.0, SPSS Inc., Armonk, NY, USA) and R software^75^. Non-parametric statistics were used since the data in the study did not show the normal distribution in the normality tests. Accordingly, we reported the EEG parameters and stroke lesion volume as the median and interquartile range in Table 1. We used the Kruskal–Wallis test to compare the EEG parameters between the control and each stroke group to determine specific stroke-related EEG features. Moreover, we used the Kruskal–Wallis test to compare stroke lesion volumes in mild, moderate, and severe stroke groups. If the results of the tests were significant, we assessed individual differences using all possible pairwise comparisons using the Mann–Whitney test with a Bonferroni correction for type I error rate inflation. Next, we conducted a univariate linear regression analysis to evaluate the relationships of the EEG parameters with stroke lesion volumes. Moreover, we calculated the coefficient of determination (R^2^) for each predictive model and the RMSE between the real and estimated stroke lesion volumes. Finally, we performed a multiple linear regression analysis with stepwise regression algorithm and assessed the multicollinearity to determine the most appropriate EEG parameter combination for estimating the stroke lesion volumes. Both the significance levels of nominal *p* value and the Bonferroni corrected *p* value were set at 0.05.

## Supplementary Information

Supplementary Information.

## Data Availability

The datasets generated and analysed during this study are publicly accessible on https://sandbox.zenodo.org/ (Digital Object Identifier: 10.5072/zenodo.680497).
