# Supplementary material for: Quantification of stroke lesion volume using epidural EEG in a cerebral ischaemic rat model
Source: Sci Rep. 2021 Jan 27;11:2308. doi: 10.1038/s41598-021-81912-2 (PMC7841185; doi:10.1038/s41598-021-81912-2)
Supplement: Supplementary file 1 — Supplementary Information. [file 41598_2021_81912_MOESM1_ESM.pdf]

Supplementary Information

Title: Quantification of stroke lesion volume using epidural EEG in a cerebral ischaemic rat model

Authors: Hyun-Joon Yoo, Jinsil Ham, Nguyen Thanh Duc, and Boreom Lee*

Supplementary Figure

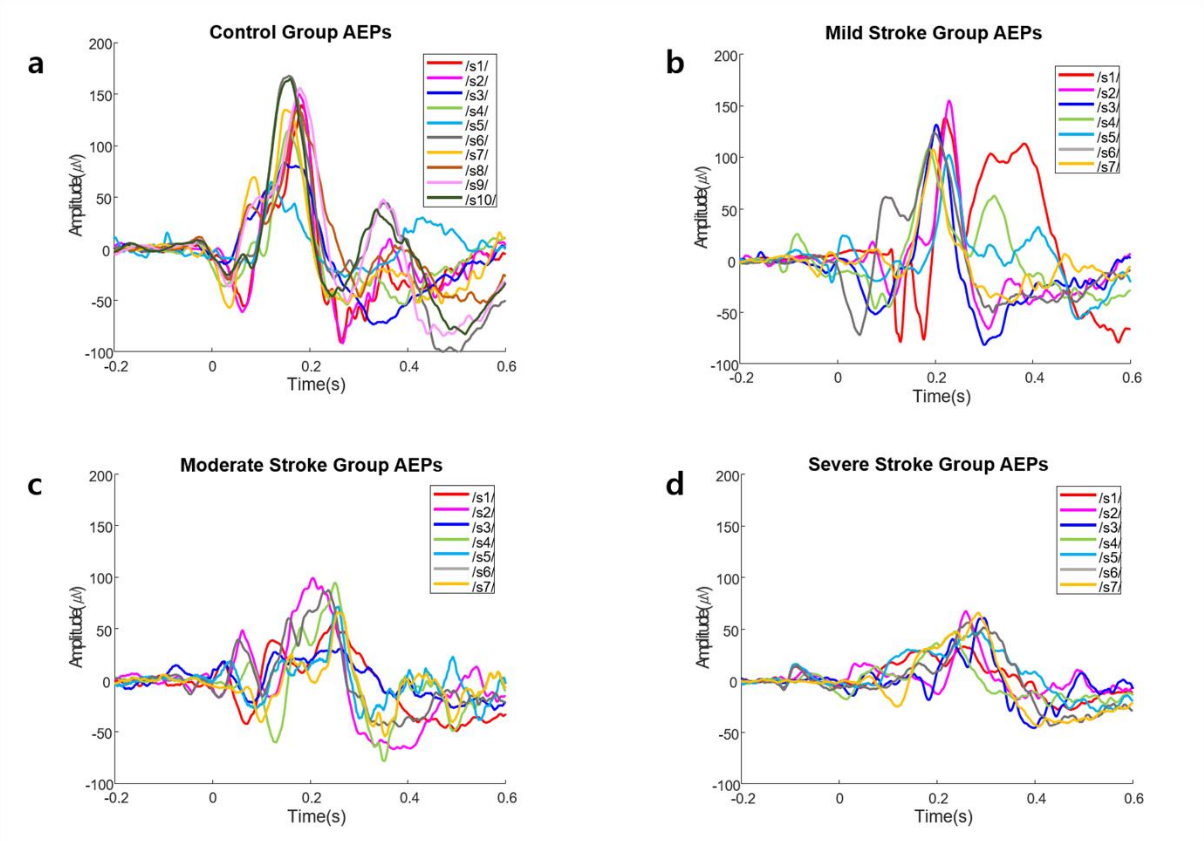

Supplementary Figure S1. Individual AEPs waveforms of each group. (a) control group, (b) mild stroke group, (c) moderate stroke group, and (d) severe stroke group. AEPs: auditory evoked potentials.

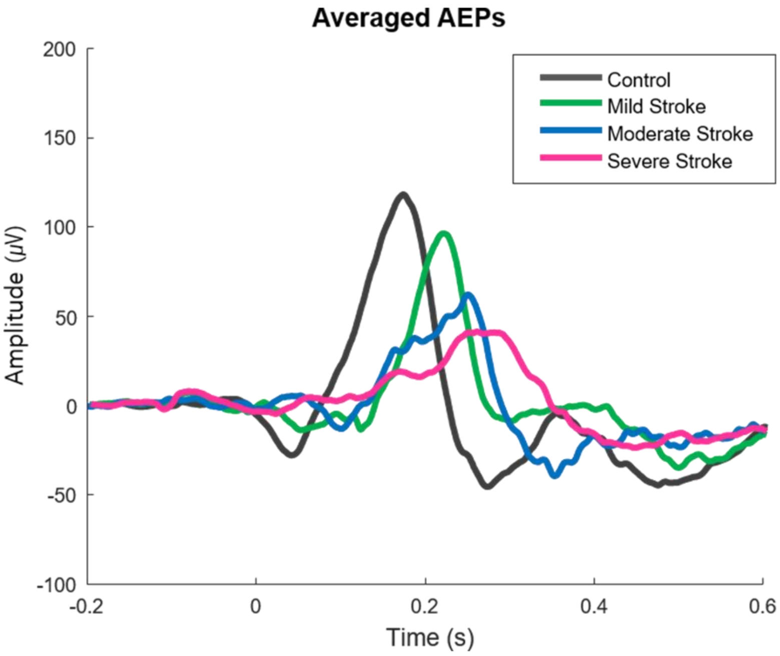

Supplementary Figure S2. Mean AEPs waveforms of each group shown in a single graph to highlight the intergroup differences. AEPs: auditory evoked potentials.

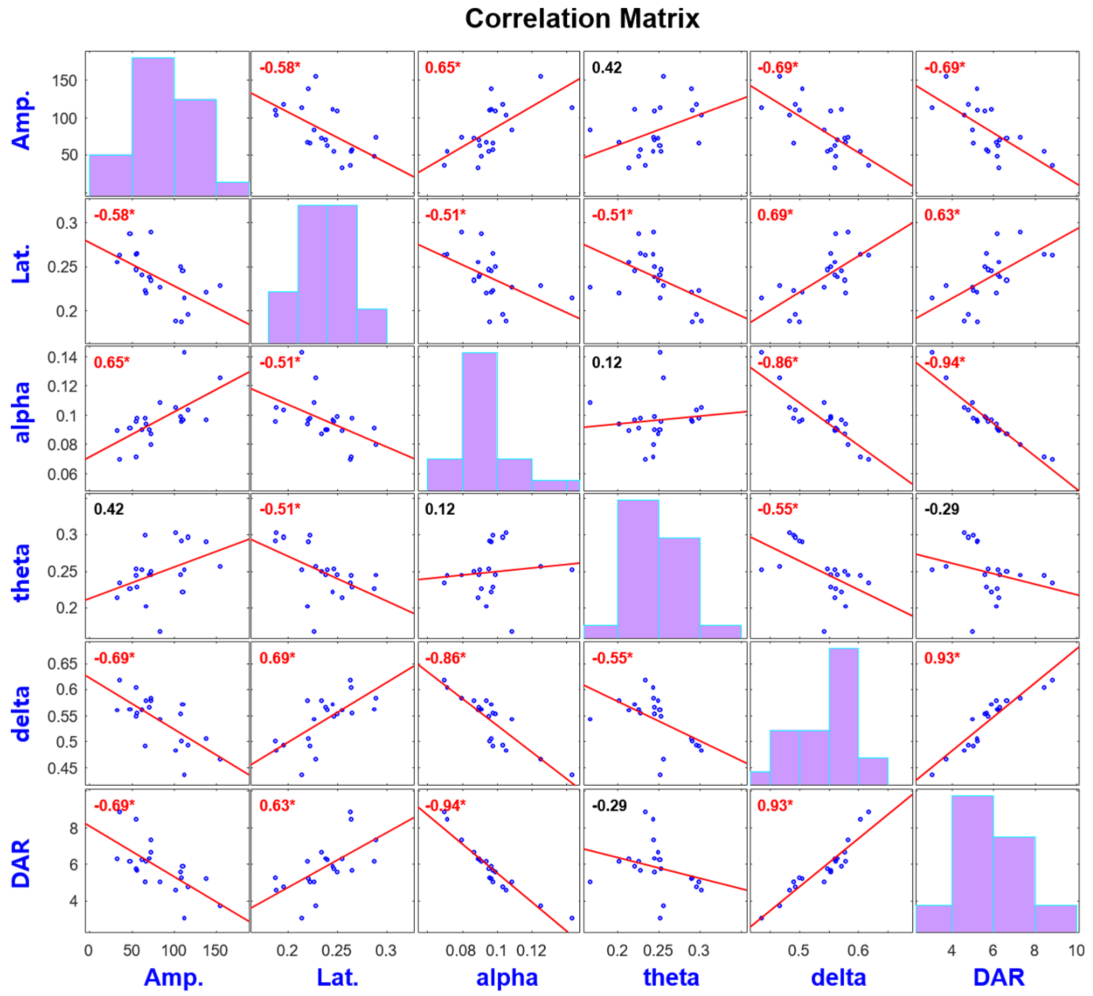

Supplementary Figure S3 Cross-correlation matrix between EEG parameters which were related to stroke lesion volume. Spearman's rank correlation coefficients are shown and the statistics with * indicate statistically significant correlations (p-value < 0.05).

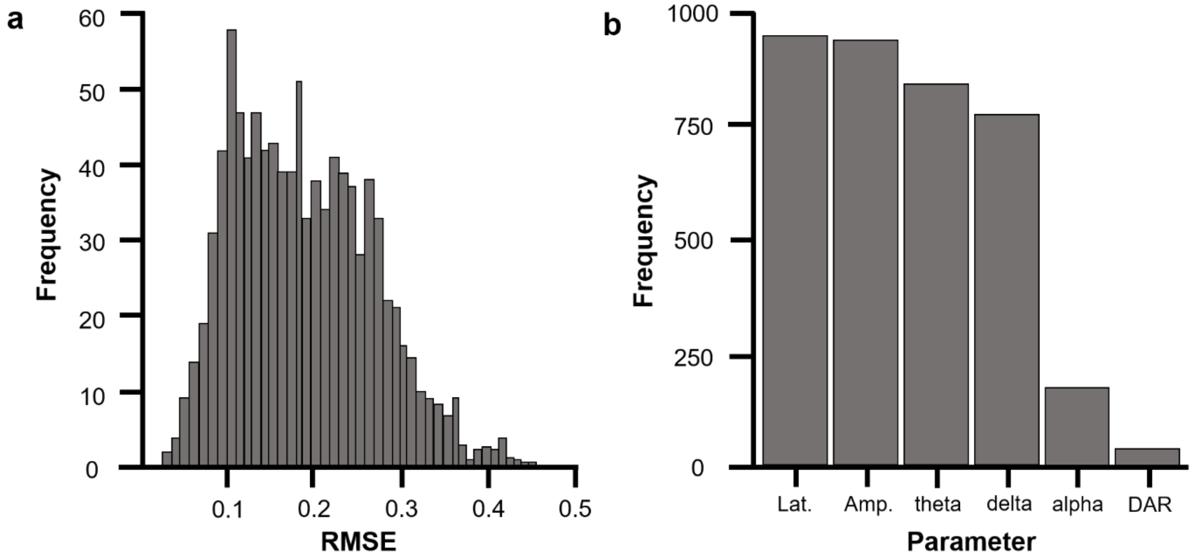

Supplementary Figure S4 Results of each multiple linear regression model using the selected training data set and test set. (a) RMSE values were calculated to evaluate the model performance, (b) the number of selected parameters on each model are shown.

Supplementary Table S1. AIC and BIC values of the linear regression models

EEG parameters	AIC	BIC
All†	-9.855	-1.450
Amplitude, Latency, RP alpha, RP theta, DAR	-11.765	-4.453
Amplitude, Latency, RP alpha, RP theta, RP delta	-11.096	-3.784
Amplitude, Latency, RP theta, RP delta, DAR	-9.534	-2.222
Amplitude, Latency, RP theta, RP delta	-13.085	-6.878
Amplitude, Latency, RP alpha, RP theta	-11.469	-5.202
Amplitude, Latency, RP theta, DAR	-9.628	-3.361
Amplitude, Latency, RP theta	-5.997	-0.775
Amplitude, Latency, RP delta	-3.543	1.679
Latency, RP alpha, RP theta	-3.010	2.213
Amplitude, Latency	2.947	7.125
Latency, RP delta	4.904	9.082
RP Delta	12.963	16.097
Latency	17.350	20.483
Amplitude	17.584	20.718
DAR	23.460	26.594
RP theta	27.227	30.361
RP alpha	28.698	31.832

The three best results of AIC and BIC based on the number of input features are shown. All†: EEG parameters represent the amplitude, the latency of AEPs and RP alpha, RP theta, RP delta, and DAR. RP, Relative power; DAR, delta/alpha ratio; DATR, (delta + theta) / (alpha + beta) ratio.
